# Morphological data and molecular characterization of *Lagostonema ecasiense* (Nematoda, Molineidae) parasite of *Lagostomus maximus* (Rodentia, Chinchillidae) from Argentina and other considerations

**DOI:** 10.1017/S0031182025100528

**Published:** 2025-08

**Authors:** Victoria Canova, María Celina Digiani, Rocío Callejón Fernández, Darío Balcazar, Natalia Beatriz Guerreiro Martins, Agustín Manuel Abba, María del Rosario del Rosario Robles

**Affiliations:** 1 Centro de Estudios Parasitológicos y de Vectores CEPAVE (CONICET-UNLP-CIC), La Plata, Buenos Aires Argentina; 2 CONICET, División Zoología Invertebrados, Facultad de Ciencias Naturales y Museo, Universidad Nacional de La Plata, La Plata, Buenos Aires Argentina; 3 Departamento de Microbiología y Parasitología de la Facultad de Farmacia, Universidad de Sevilla, Sevilla, España

**Keywords:** Argentina, helminth, Plains viscacha, rodent

## Abstract

*Lagostonema ecasiense* is a bursate nematode parasite of *Lagostomus maximus* in Argentina. New morphological data, geographical distribution, ecological data, molecular characterization and exploratory phylogenetic analysis are provided. The general morphology and measurements agree with the original description with minimal discrepancies. The geographical distribution of *Lagostonema* is expanded with 3 new provinces and 9 new departments in Argentina. The molecular characterization constitutes the first molecular contribution for the genus *Lagostonema*. The analysis of genetic distances and phylogenetic exploration allow considering *L. ecasiense* as a nominal species, confirming its nomenclatural taxonomic identity. Likewise, although morphological studies allow the identification of specimens from all populations as *L. ecasiense*, molecular studies show a major genetic distance in the population from Santiago del Estero Province concerning the rest of the populations. Consequently, the haplotypes are mentioned as *Lagostonema* sp. with the possibility that these specimens belong to a new species. This study is valuable because it contributes to the ratification of a nominal species described decades ago, adding new morphological aspects and providing an understanding of their value as a marker of host populations.

## Graphical abstract

**Figure:**
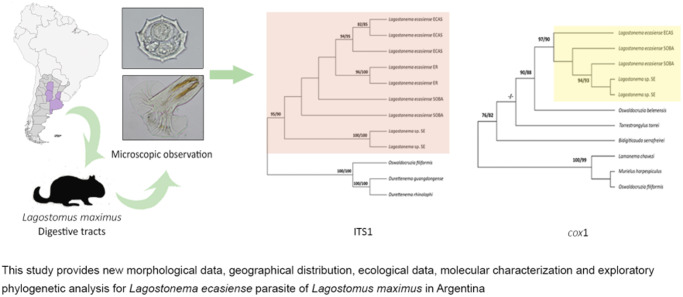

## Introduction

Molineidae Durette-Desset and Chabaud, 1977 (Nematoda, Molineoidea) is a family of bursate nematodes that is composed of 6 subfamilies: Molineinae Skrjabin and Schulz, 1937, Dromaeostrongylinae (Skrjabin and Schulz, 1937), Mertensinematinae Sharpilo, 1976, Nematodirinae Skrjabin and Orloff, 1934, Anoplostrongylinae Chandler, 1938 and Ollulaninae Hall, 1916 (Beveridge et al., 2014). These subfamilies are mainly differentiated by the synlophe, tail with or without spine or tubercles in the females, and the shape of the dorsal ray of the caudal bursa, and are composed of species that parasitize a wide variety of vertebrates (Durette-Desset, 1985; Durette-Desset et al., 1999; Beveridge et al., 2014). According to Beveridge et al. (2014), the subfamily Molineinae is characterized by a cephalic vesicle, synlophe with ridges oriented perpendicularly to the body surface, caudal bursa of type 2-3 tending to 2-1-2 or 3-2, and females didelphic, oviparous and generally with a caudal spine. This subfamily is composed of 28 genera of parasites with a cosmopolitan distribution that parasitize amphibians, reptiles and mammals (Beveridge et al., 2014; Ju et al., 2017; Guerrero, 2020).

*Lagostonema* Sutton and Durette-Desset, 1987 is a monospecific genus, being the type species *Lagostonema ecasiense* Sutton and Durette-Desset, 1987, a parasite of *Lagostomus maximus* (Desmarest, 1817) (Rodentia, Chinchillidae) from a semi-captive population hosted in a Wildlife Park named Estación de Cría de Animales Silvestres (ECAS), Berazategui Department, Buenos Aires Province. At present, *L. ecasiense* has been recorded only in Buenos Aires (Sutton and Durette-Desset, 1987; Canova et al., 2024) and La Pampa provinces (Foster et al., 2002) from this same host species.

*Lagostomus maximus* is a medium-sized herbivorous species, endemic to South America and inhabiting central, eastern and northern Argentina, western Paraguay and southeastern Bolivia. This rodent presents semi-fossorial, nocturnal and highly gregarious habits (Jackson et al., 1996; Spotorno and Patton, 2015). This mammal species was declared a national agricultural pest in 1905 due to the damage caused by its burrows and competition with livestock for food, which led to official campaigns for its control and eradication. Furthermore, it has been hunted for commercial (meat and fur) and sporting purposes. As a result, the species has disappeared from several areas across its range. Despite this, its conservation status is currently listed as Least Concern due to its wide geographic distribution, presence in protected areas and ability to occupy human-modified areas. Currently, there are hunting regulations in several parts of its geographic distribution (Jackson et al., 1996; Cirignoli and Lartigau, 2019).

The aim of this paper was to deepen knowledge about *L. ecasiense* of different populations of *L. maximus* from Argentina, contributing with (1) new morphological data, (2) geographical distribution, (3) ecological data and (4) molecular characterization with 2 molecular markers (internal transcribed spacer 1 -ITS1- ribosomal DNA and *cytochrome c oxidase* I -*cox*1- mitochondrial DNA) and an exploratory phylogenetic analysis.

## Materials and methods

### Material examined

**Figure 1.**
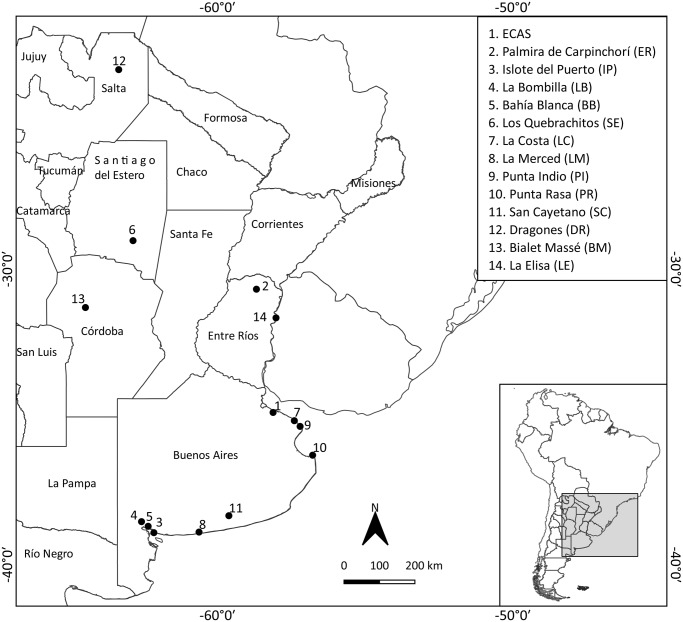
Map of Argentina with sites of origin of *Lagostomus maximus* specimens studied. See data of localities in Table 1.

A total of 70 digestive tracts belonging to *L. maximus* (including stomach, small intestine, large intestine, pancreas and liver) were examined for parasites. Fifteen specimens belonging to the Mammals Collection of Museo Argentino de Ciencias Naturales Bernardino Rivadavia (MACN) from 4 provinces of Argentina: Buenos Aires: San Cayetano (SC, *n* = 6), Salta: Dragones (DR, *n* = 1), Córdoba: Bialet Massé (BM, *n* = 3) and Entre Ríos: La Elisa (LE, *n* = 5). Fifty-five specimens were collected between 2012 and 2021 from 10 sites that belong to 3 provinces of Argentina: Buenos Aires (*n* = 30): Campo La Costa (LC, *n* = 1), Bahía Blanca (BB, *n* = 2), Establecimiento La Merced (LM, *n* = 2), ECAS (*n* = 12), Punta Indio (PI, *n* = 1), Punta Rasa (PR, *n* = 1), Islote del Puerto (IP, *n* = 1), Campo La Bombilla (LB, *n* = 10); Entre Ríos: Estancia Palmira de Carpinchorí (ER, *n* = 12); and Santiago del Estero: Estancia Los Quebrachitos (SE, *n* = 13) (Table 1, Figure 1). Bahía Blanca, Islote del Puerto and Campo La Bombilla were brought together under the acronym Southwest of the Province of Buenos Aires (SOBA).

**Table 1.** Detail of *Lagostomus maximus* specimens examined from Argentina and ecological data on *Lagostonema* specimens

Map code	Locality/site	Coordinates	Department	Date	Ecological data		
					P (%)	MI	MA
Housed at MACN^a^ (*n* = 15)							
11	SC	38°20ʹ35.66″S, 59°36ʹ41.21″W	San Cayetano	1936	1/6	–	–
12	DR	23°14ʹ55.56″S, 63°20ʹ25.39″W	Gral. José de San Martín	1936	Not parasitized		
13	BM	31°17ʹ54.42″S, 64°28ʹ0.12″W	Punilla	1939	1/3	–	–
14	LE	31°38ʹ53.54″S, 58°0ʹ55.45″W	Concordia	1948 and 1949	4/5	–	–
Specimens sampled for this study							
Buenos Aires Province (*n* = 30)					96.7	59.7	57.7
7	LC	35°10ʹ35.43″S, 57°20ʹ47.65″W	Magdalena	2012	100 (1/1)	77	77
5	BB^b^	38°42ʹ4.38″S, 62°20ʹ37.85″W	Bahía Blanca	2015 and 2019	100 (2/2)	24.5	24.5
8	LM	38°53ʹ50.00″S, 60°37ʹ17.00″W	Tres Arroyos	2017	100 (2/2)	99.5	99.5
1	ECAS	34°50ʹ58.32″S, 58°6ʹ52.72″W	Berazategui	2017 and 2018	100 (12/12)	53.9	53.9
9	PI	35°16ʹ23.95″S, 57°14ʹ52.39″W	Punta Indio	2018	100 (1/1)	7	7
10	PR	36°17ʹ44.30″S, 56°46ʹ32.96″W	La Costa	2019	100 (1/1)	62	62
3	IP^b^	38°49ʹ15.80″S, 62°16ʹ13.40″W	Bahía Blanca	2019	100 (1/1)	126	126
4	LB^b^	38°32ʹ39.95″S, 62°34ʹ6.68″W	Tornquist	2019	90 (9/10)	62.7	56.4
Entre Ríos Province (*n* = 12)							
2	ER	30°40ʹ43.06″S, 58°40ʹ40.61″W	Federal	2018	100 (12/12)	173.3	173.3
Santiago del Estero Province (*n* = 13)							
6	SE	29°2ʹ7.90″S, 62°51ʹ19.80″W	Aguirre	2021	100 (13/13)	313.8	313.8

a MACN = Mammals Collection from Museo Argentino de Ciencias Naturales Bernardino Rivadavia.

b Sites of southwest of Buenos Aires Province (SOBA).

The digestive tracts were fixed in 10% formalin or preserved in 96% ethanol, and nematodes were localized under a stereo-microscope (Olympus SZ61-TR), collected and preserved in 70% or 96% ethanol for morphological and molecular studies.

### Morphological analysis

A total of 62 *Lagostonema* specimens from 13 sites (Table 1) were cleared in lactophenol and studied under light microscopy (Olympus BX51). Drawings were made with the aid of a drawing tube. Measurements were recorded in micrometres (μm) and expressed as mean, standard deviation and range between parentheses. These measurements were compared with those published by Sutton and Durette-Desset (1987) (Table 2). On the other hand, transverse sections obtained along the body of 18 *Lagostonema* specimens were mounted to study the synlophe under light microscopy (Olympus BX51). Transverse body sections were illustrated with the dorsal side of the worm oriented to the top of the page. Voucher specimens were deposited in the Helminthological Collection of Museo de La Plata (MLP-He), La Plata, Buenos Aires, Argentina.

**Table 2.** Main morphological features and measurements of *Lagostonema* specimens; measurements are presented in micrometres as mean values and standard deviations followed by range values in parentheses

Species	*Lagostonema ecasiense*		*Lagostonema ecasiense*		*Lagostonema* sp.	
Reference	Sutton and Durette-Desset (1987)		This paper		This paper	
Host	*Lagostomus maximus*		*Lagostomus maximus*		*Lagostomus maximus*	
Localities	ECAS, Berazategui, Buenos Aires		ECAS, SOBA and ER		SE	
	Male	Female	Male	Female	Male	Female
	1 (holotype)	1 (allotype)	21	21	10	10
Body length (BL)	6200	10200	5126 ± 716 (4120*–*6430)	6561 ± 748 (5341*–*8402)	7226 ± 801 (5703*–*8193)	9101 ± 1209 (6999*–*11147)
Body width (BW)	90	105	70 ± 7 (55*–*93)^a^	69 ± 7 (56*–*83)^a^	84 ± 9 (70*–*100)^a^	93 ± 27 (71*–*163)^a^
Cephalic vesicle length	75	80	69 ± 8 (53*–*85)^b^	72 ± 14 (50*–*116)^f^	79 ± 10 (63*–*98)	74 ± 12 (61*–*92)
Cephalic vesicle width	40	30	34 ± 3 (27*–*39)^b^	37 ± 8 (30*–*68)^f^	39 ± 4 (33*–*45)	43 ± 2 (37*–*45)
Nerve ring (dfae)	200	185	169 ± 27 (113*–*249)	164 ± 19 (132*–*203)^b^	180 ± 22 (147*–*210)	176 ± 33 (123*–*231)
Excretory pore (dfae) (EP)	415	400	299 ± 48 (225*–*378)^c^	314 ± 63 (203*–*397)^g^	378 ± 46 (316*–*439)^k^	340 ± 50 (267*–*412)^k^
Deirids (dfae)	420	410	308 ± 48 (234*–*388)^b^	316 ± 67 (210*–*392)^h^	389 ± 41 (338*–*446)^k^	359 ± 45 (306*–*406)^m^
Oesophagus total length	375	420	357 ± 24 (308*–*398)	370 ± 18 (337*–*401)	373 ± 24 (336*–*413)	399 ± 30 (360*–*442)
Spicule length (SL)	140		124 ± 10 (110*–*151)^d^		138 ± 9 (124*–*151)	
Gubernaculum length	90		73 ± 8 (61*–*87)^e^		78 ± 9 (69*–*94)^l^	
Vulva (dfpe)		2300		1438 ± 193 (1111*–*1815)		2145 ± 333 (1579*–*2702)
Vagina vera		40		33 ± 5 (25*–*44)^e^		38 ± 11 (26*–*60)
Ovejector length		830		722 ± 64 (592*–*829)^d^		862 ± 95 (765*–*1019)^k^
Vestibule		455		387 ± 50 (305*–*494)		475 ± 66 (406*–*608)
Anterior sphincter minor axis		55		41 ± 7 (30*–*56)		53 ± 5 (44*–*59)
Anterior sphincter major axis		60		45 ± 5 (37*–*52)		56 ± 6 (46*–*66)
Anterior infundibulum length		140		133 ± 20 (106*–*192)^c^		143 ± 18 (112*–*174)^k^
Posterior sphincter minor axis		50		43 ± 10 (27*–*66)		51 ± 5 (42*–*57)
Posterior sphincter major axis		65		46 ± 5 (37*–*58)		56 ± 5 (49*–*67)
Posterior infundibulum length		130		124 ± 16 (96*–*154)^f^		135 ± 16 (114*–*156)^l^
Anterior uterine branch (number of eggs)		19		10 ± 3 (6*–*16)^i^		19 ± 5 (14*–*25)^n^
Posterior uterine branch (number of eggs)		13		7 ± 2 (5*–*12)^i^		16 ± 6 (9*–*24)^n^
Tail length (TL)		130		85 ± 12 (65*–*111)^b^		108 ± 8 (96*–*123)^k^
Eggs width (EW)		45		38 ± 3 (32*–*46)^j^		40 ± 5 (33*–*55)^o^
Eggs length (EL)		75		65 ± 5 (55*–*78)^j^		62 ± 4 (51*–*70)^o^
BW/BL (%)	1.4	1.03	1.4 ± 0.2 (1.1*–*1.7)	1.1 ± 0.1 (0.9*–*1.2)	1.2 ± 0.1 (1*–*1.4)	1 ± 0.3 (0.8*–*1.9)
EP/BL (%)	6.7	3.9	5.8 ± 1 (4.1*–*8.9)^c^	4.6 ± 0.9 (2.8*–*5.7)^g^	5.1 ± 0.5 (4.3*–*6)^k^	3.6 ± 0.5 (2.9*–*4.2)^k^
SL/BL (%)	2.2		2.5 ± 0.3 (1.9*–*3.1)^d^		1.9 ± 0.2 (1.7*–*2.2)	
Vulva/BL (%)		22.5		21.9 ± 1.5 (19.1*–*24.9)		23.5 ± 0.9 (21.9*–*24.5)
TL/BL (%)		1.3		1.3 ± 0.2 (1*–*1.8)^b^		1.2 ± 0.1 (1*–*1.4)^k^
EW/EL (%)		60		58.2 ± 5.9 (46.7*–*74.5)^j^		64.8 ± 7.2 (54*–*87.9)^o^

dfae: distance from anterior end; dfpe: distance from posterior end.

a Width at midbody.

b *n* = 20.

c *n* = 19.

d *n* = 17.

e *n* = 16.

f *n* = 18.

g *n* = 13.

h *n* = 11.

i *n* = 15.

j *n* = 105.

k *n* = 8.

l *n* = 9.

m *n* = 7.

n *n* = 5.

o *n* = 50.

A principal component analysis (PCA) was performed to explore the morphometric characteristics of *Lagostonema* specimens from different host populations (SOBA, ER, ECAS and SE). The analysis was carried out using the ggplot2 package R (R Core Team, 2022). A total of 62 specimens (31 males and 31 females) and 22 morphometric variables (11 for males and 11 for females) were included.

### Molecular and phylogenetic analysis

Fourteen *Lagostonema* specimens stored in 96% ethanol and previously identified on morphological traits were used for the polymerase chain reaction (PCR) and sequencing. Genomic DNA from individual worms was extracted and purified using a commercial DNA extraction kit (Wizard® Genomic DNA Purification Kit, Promega, Madison, WI, USA) according to the manufacturer’s protocol for tissues, to 2 genomic fragments: ITS1 (to 9 specimens from ECAS, SOBA, ER and SE) and *cox*1 (to 5 specimens from ECAS, SOBA and SE). All the amplifications were performed in a Multigene Labnet International, Inc. thermocycler and the following mix: each 50 μL PCR contained 1× GoTaq Green Master Mix (Promega, Madison, WI), 0.4 μM of each primer and 1 μL of the extracted DNA. Fragments of ITS1 were amplified using the primers AngioF1674 (forward) and 58SR4 (reverse) described by Qvarnstrom et al. (2010), with the following conditions: initial denaturation at 94°C for 5 min, 35 cycles of 94°C for 30 s, 54°C for 30 s and 72°C for 1 min, and final extension at 72°C for 3 min. Regarding *cox*1 gene marker fragments were amplified using the primers LCO 1490 (forward) and HCO 2198 (reverse) described by Sharma and Kobayashi (2014), with the following conditions: initial denaturation at 94°C for 5 min, 35 cycles of 94°C for 30 s, 46°C for 40 s and 72°C for 1 min, and final extension at 72°C for 10 min. The PCR products were checked on ethidium bromide-stained 1.5% Tris-Borate-EDTA using 0.8% agarose gels electrophoresis and examined by UV transillumination. All PCR products were purified and sequenced in both directions with amplifying primers (Macrogen, Seoul, Korea).

Consensus sequences were constructed and compared using the algorithm BLASTn to known sequences from GenBank, the National Center for Biotechnology Information database (https://www.ncbi.nlm.nih.gov). To obtain aligned sequences, the MUSCLE alignment method (Edgar, 2004) was used by MEGA 5 version 5.2 program (Tamura et al., 2011). To evaluate the similarity between different species obtained from GenBank, the number of differences between bases was analysed using the MEGA 5 version 5.2 program (Tamura et al., 2011). Phylogenetic trees were generated with 2 methods: Maximum Likelihood (ML) and Bayesian Inference (BI), using the PhyML package (Guindon and Gascuel, 2003), MEGA 5 program (Tamura et al., 2011) and MrBayes version 3.1.2 (Ronquist and Huelsenbeck, 2003), respectively. Species of Molineinae and other Molineidae available in GenBank were used as outgroups (Table 3).

**Table 3.** Sequences of *Lagostonema* specimens and other Molineinae/Molineidae species used for phylogenetic analyses (GenBank accession numbers)

Gene/region	Species	Host species	Geographical origin	GenBank accession numbers
ITS1	*Lagostonema ecasiense*	*Lagostomus maximus*	Argentina	
			ECAS	PP028739
			ECAS	PP028740
			ER	PP028741
			ER	PP028742
			SOBA	PP028743
			SOBA	PP028744
			ECAS	PP028745
	*Lagostonema* sp.	*Lagostomus maximus*	SE	PP028746
			SE	PP028747
	*Oswaldocruzia filiformis*	*Lissotriton vulgaris*	Rhineland-Palatinate, Germany	MG586088.1
	*Durettenema guangdongense*	*Hipposideros larvatus*	Longmen, Guangdong Province, China	KX856930.1
	*Durettenema rhinolophi*	*Scotophilus kuhlii*	–	KX912249.1
*cox*1	*Lagostonema ecasiense*	*Lagostomus maximus*	Argentina	
			ECAS	OR981615
			SOBA	OR981616
			BB	OR981617
	*Lagostonema* sp.	*Lagostomus maximus*	SE	OR981618
			SE	OR981619
	*Oswaldocruzia filiformis*	*Rana arvalis*	Orenburg region, Russia	MT300256.1
	*Oswaldocruzia belenensis*	*Rhinella* gr. *margaritifera*	Pará State, Brazil	MK492915.1
	*Torrestrongylus torrei*	*Macrotus waterhousii*	Mexico	KF425294.1
	*Bidigiticauda serrafreirei*	*Artibeus planirostris*	Bahia State, Brazil	MF375800.1
	*Lamanema chavezi*	*Lama glama*	Salta Province, Argentina	MG598415.1
	*Murielus harpespiculus*	*Ochotona princeps*	Roman Nose Lakes, Idaho, USA	KP876338.1

### Data analysis

Ecological parameters including prevalence (P), mean intensity (MI) and mean abundance (MA) were calculated for hosts and provinces (specimens housed at MACN were excluded) according to Bush et al. (1997).

## Results

### Morphological analysis

Of a total of 70 *L. maximus* obtained for the parasitological survey, 60 were parasitized with *Lagostonema* specimens: 6 of 15 from MACN and 54 of 55 from field samples (Table 1).

A total of 8007 specimens of *Lagostonema* were recovered from the stomach and small intestine, being much more abundant in this latter (Table 4). Among these, 42 specimens of *L. ecasiense*: 28 from Buenos Aires Province and 14 from Entre Ríos Province, and 20 specimens of *Lagostonema* sp. from Santiago del Estero Province were measured (Table 2).

**Table 4.** Mean abundance of *Lagostonema* specimens in stomach and small intestine in *Lagostomus maximus* from sampled provinces

Province	Stomach			Small intestine		
	Average	s.d.	Range	Average	s.d.	Range
Buenos Aires	2.2	3.9	0*–*17	55.3	74.4	0*–*378
Entre Ríos	3.8	7.3	0*–*21	169.4	145.8	1*–*412
Santiago del Estero	2.1	5.8	0*–*21	310.8	308.4	67*–*1143

s.d.: standard deviation.

## Redescription

### Lagostonema ecasiense Sutton and Durette-Desset, 1987

**Figure 2.**
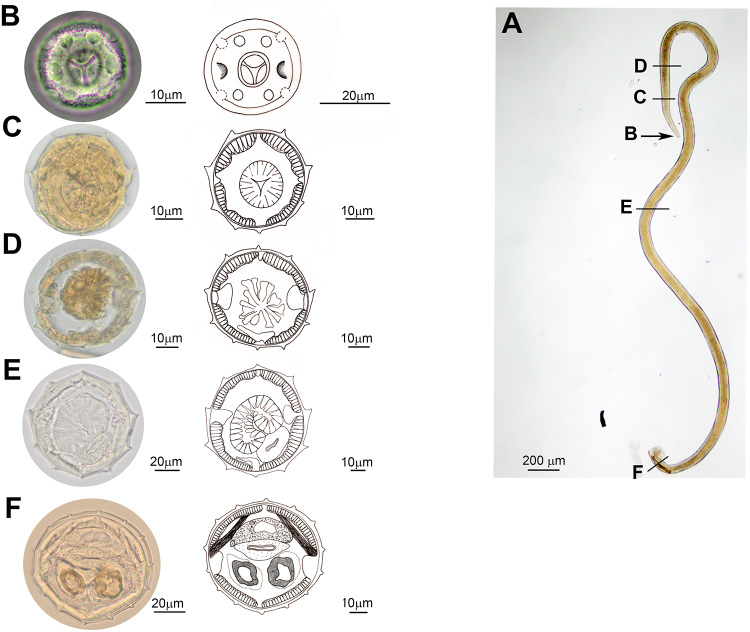
Light microscope photographs and drawings of *Lagostonema ecasiense*. Male. (A) Whole specimen with indications of the apical view (B) and the transverse sections made to study the synlophe (C–F). (B) Anterior end, apical view. (C–F) Cross sections of the synlophe in different regions of the body: (C) at oesophagus level, (D) at oesophago–intestinal junction, (E) at midbody, (F) in front of caudal bursa.

*General diagnosis*: Small nematodes with body slightly coiled or not coiled. Cephalic vesicle present. *In apical view*: lips absent, buccal aperture rounded, 2 amphids, 4 external labial papillae and 4 cephalic papillae observed (Figure 2B). Excretory pore and deirids situated at approximately the same level, slightly in front of the end of the oesophagus (Figure 4E).

**Figure 3.**
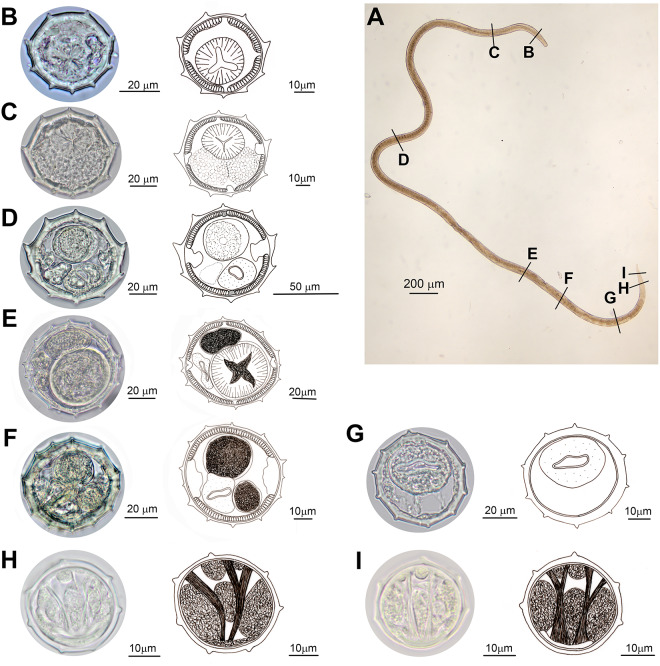
Light microscope photographs and drawings of *Lagostonema ecasiense*. Female. (A) Whole specimen with indications of level of transverse sections made to study the synlophe. (B–I) Cross sections of the synlophe in different regions of the body: (B) at oesophagus level, (C) at oesophago–intestinal junction, (D) at midbody, (E) before vulva (anterior infundibulum), (F) behind vulva (posterior uterine branch), (G) distal part of intestine, (H–I) tail.

*Synlophes (based on 10 males and 8 females)*: body with continuous cuticular ridges without struts, extending along the entire body with an axis of orientation coincident with the sagittal axis and directed from ventral to dorsal (Figures 2, 3).

In both sexes, synlophe bears 9 ridges at level of muscular oesophagus, and 10 ridges both at level of oesophago–intestinal junction and at midbody, with 3 dorsal ridges, 5 ventral ridges and 2 lateral ridges (associated to the 2 lateral hypodermal cords) more developed (Figures 2C–E, 3B–D). In males, number of ridges increases progressively until reaching 15 (range: 12–15) in front of the caudal bursa (Figure 2F). In females, number of ridges increases progressively until reaching 14 at level of vulva; then progressively decreases to 5 ridges at level of tail (Figure 3E–I). In both sexes, behind midbody, lateral ridges decrease in size (Figures 2F, 3E–I).

**Figure 4.**
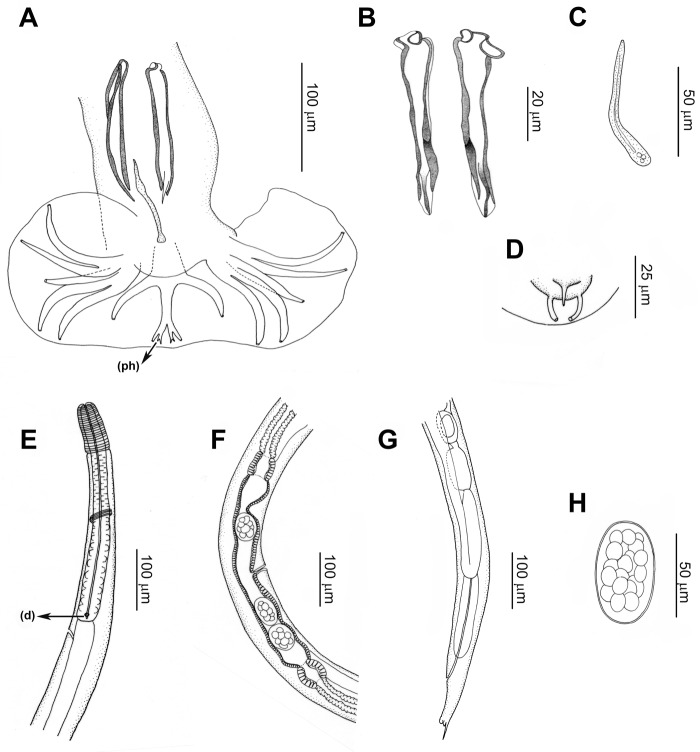
*Lagostonema ecasiense*. Male. (A) Caudal bursa, ventral view, the arrow indicates one phasmid (ph). (B) Spicules. (C) Gubernaculum. (D) Genital cone, ventral view. Female. (E) Anterior end, left lateral view, the arrow indicates the deirid (d). (F) Ovejector, right lateral view. (G) Posterior end, left lateral view. (H) egg.

*Males (based on 31 specimens)*: The measurements are shown in Table 2. Caudal bursa subsymmetrical of type 2-1-2 with small dorsal lobe. Rays 2 the longest. Rays 4 very short. Rays 8 not reaching the extremities of the dorsal ray and arising from the base of this latter (Figure 4A). Dorsal ray divided into 2 sub-branches at its extremity, each sub-branch bearing 3 papillae: papilla 9 (external), papilla 10 (internal) and phasmid (middle). Subequal alate spicules divided into 3 points at their distal end, the latero-dorsal point being the longest (Figure 4B). Gubernaculum enlarged at its distal end and curved ventrally (Figure 4C). Genital cone bearing on its ventral lip the papilla zero and on its dorsal lip 2 rod-like papillae 7 (Figure 4D).

*Females (based on 31 specimens*): The measurements are shown in Table 2. Didelphic (Figure 4F). Vulva in the posterior quarter of the body. Ovejector symmetrical (Figure 4F). Tail with distal spine and 2 sublateral tubercles (Figure 4G). Eggs oval, with thin shell, some morulated and other in gastrulation stage (Figure 4H).

## Taxonomic summary

### Host:

Lagostomus maximus *(Desmarest, 1817)*: Localities: specimens measured were from ECAS, SOBA, ER and SE (Table 2, the specimens from SE are refered as *Lagostonema* sp. in the table). Other specimens studied were from Buenos Aires Province: SC, LC, LM, PI and PR; Córdoba Province: BM; and Entre Ríos Province: LE.

Site of infection: small intestine and, secondarily, stomach.

Specimens deposited: MLP-He 8073, MLP-He 8074, MLP-He 8075, MLP-He 8076, MLP-He 8077, MLP-He 8078, MLP-He 8079 and MLP-He 8080.

Ecological data: The highest values of total P were registered for ER and SE. The highest values of MI and MA were registered for SE (Table 1).

## Remarks

*Lagostonema ecasiense* was described based on 1 male (holotype) and 1 female (allotype) (Sutton and Durette-Desset, 1987). In the present work, several specimens were studied. The general morphology and measurements examined agree with those studied by Sutton and Durette-Desset (1987), with minimal discrepancies in females: e.g. body width (105 vs 56–83), cephalic vesicle width (30 vs 30–68), distance from anterior end to deirids (410 vs 210–392), posterior sphincter major axis (65 vs 37–58) and tail length (130 vs 65–111) (Table 2). The *Lagostonema* sp. specimens from SE remain the same morphological considerations described to *Lagostonema ecasiense*, with minimal metric variations observed in Table 2.

On the other hand, in the original description, the synlophe was only described at midbody. In this study, slight variation in the number and size of the ridges of the synlophe throughout the body and between males and females was observed (Figures 2, 3).

### Morphometric analysis

The PCA conducted to explore the morphometric characteristics between the *Lagostonema* specimens did not reveal groupings among the host populations studied (SOBA, ER, ECAS and SE), although a slight tendency towards separation of the SE specimens can be interpreted. In this sense, the ranges of most diagnostic characters (e.g., ratios such as BW/BL, EP/BL, SL/BL, Vulva/BL and TL/BL) are overlapped (Table 2, Supplementary Fig. S1).

### Molecular and phylogenetic studies

Nucleotide sequence data of the rDNA (ITS1) and mtDNA (*cox*1) are reported and are available in GenBank (GenBank accession number, Table 3). The rDNA (ITS1) revealed 9 haplotypes, and their sequences were 528–534 base pairs (bp) (exclusive of the primers); G + C content ranged between 44% and 45%, while A + T content ranged between 29.9% and 31.5%. Multiple alignments of 12 ITS1 sequences from species in the subfamily Molineinae produced a data set of 549 characters. The maximum and minimum values of similarity among studied populations were observed between the isolates from ECAS and ER (98.36–99.45%), and ER/SOBA and SE (79.78–83.78% and 79.60–84.69%), respectively. The minimum and maximum values between the *Lagostonema* studied populations and the other 3 species studied were 63.20% concerning *Durettenema* spp. and 82.51% concerning *Oswaldocruzia filiformis* (Table 5).

**Table 5.** Intra- and inter-specific similarity percentage observed with ITS1 rDNA between *Lagostonema* specimens from different *Lagostomus maximus* populations and other species of Molineinae

	*L. ecasiense* ECAS	*L. ecasiense* ER	*L. ecasiense* SOBA	*Lagostonema* sp. SE	*Durettenema guangdongense*	*Durettenema rhinolophi*	*Oswaldocruzia filiformis*
*L. ecasiense* ECAS	99.64						
*L. ecasiense* ER	98.36*–*99.45^a^	99.87					
*L. ecasiense* SOBA	80.19*–*95.63	94.35*–*95.81	92.89				
*Lagostonema* sp. SE	80.51*–*80.87	79.78*–*83.78^b^	79.60*–*84.69^b^	85.61			
*D. guangdongense*	74.13*–*74.32	73.40	72.31*–*73.58	63.20^c^*–*66.12	–		
*D. rhinolophi*	74.13*–*74.31	73.41	72.31*–*73.59	63.20^c^*–*66.12	99.81	–	
*O. filiformis*	82.33	82.33*–*82.51^d^	79.41*–*80.15	70.13*–*72.31	74.31	74.50	–

a Maximum value of similarity among *Lagostonema* studied populations.

b Minimum value of similarity among *Lagostonema* studied populations.

c Minimum value between *Lagostonema* studied populations and other Molineinae species.

d Maximum value between *Lagostonema* studied populations and other Molineinae species.

**Figure 5.**
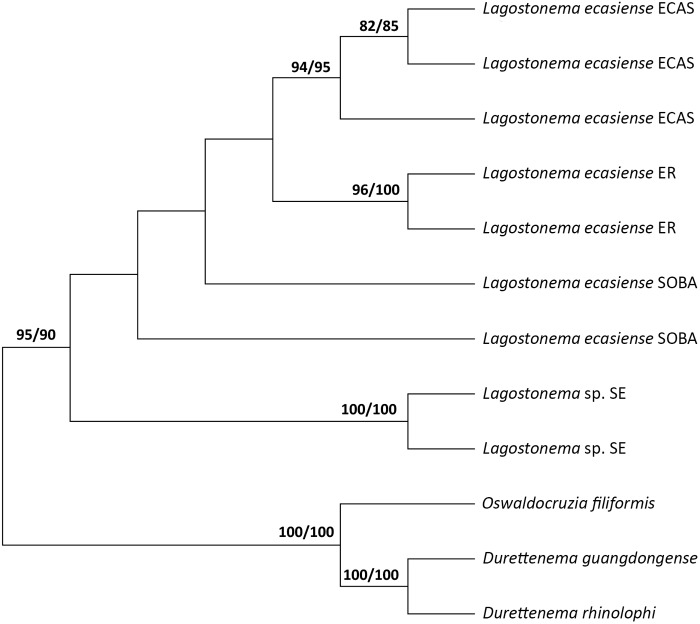
Phylogenetic tree of Molineinae species based on ITS1 rDNA region obtained with Maximum Likelihood (ML) and Bayesian Inference (BI).

The consensus tree showed 2 phylogenetic groups corresponding to genus *Lagostonema* and the 3 species of the Molineinae analysed, with good resolution (95 BI/90 ML). Considering the analysed populations of *Lagostonema* specimens, the formation of at least 2 robust clades was observed, corresponding to the haplotypes of ECAS (94 BI/95 ML) and ER (96 BI/100 ML). The position of the haplotypes from the southwest of Buenos Aires Province (SOBA) was unclear, but they were separated from the other subclades. In contrast, SE specimens formed a separate clade, clearly distinct from the other populations with high resolution (100 BI/100 ML) (Figure 5).

The mtDNA (*cox*1) encoding gene revealed 5 haplotypes and their sequences were of 337 bp (exclusive of the primers); G + C content ranged between 29.9% and 31.5%, while the A + T content ranged between 67.9% and 70.1%. Multiple alignments of 11 *cox*1 sequences from species in the family Molineidae produced a data set of 337 characters. The maximum and minimum values of similarity between populations were observed between the isolates from SOBA and SE (98.51%), and SOBA and ECAS (88.72%–89.31%), respectively. The minimum and maximum values between *Lagostonema* specimens and the studied species belonging to other genera were 45.40% concerning *Oswaldocruzia filiformis* and 89.91% concerning *Oswaldocruzia belenensis*, respectively (Table 6).

**Table 6.** Intra- and inter-specific similarity percentage observed with *cox*1 mtDNA between *Lagostonema* specimens from different *Lagostomus maximus* populations and other species of Molineidae

	*L. ecasiense* ECAS	*L. ecasiense* SOBA	*Lagostonema* sp. SE	*Oswaldocruzia belenensis*	*Oswaldocruzia filiformis*	*Bidigiticauda serrafreirei*	*Torrestrongylus torrei*	*Lamanema chavezi*	*Murielus harpespiculus*
*L. ecasiense* ECAS	–								
*L. ecasiense* SOBA	88.72*–*89.31^a^	98.22							
*Lagostonema* sp. SE	89.02*–*89.20	98.51^b^	100						
*O. belenensis*	87.24	89.31	89.91^c^	–					
*O. filiformis*	45.40^d^	48.07*–*48.36	48.36*–*48.37	49.26	–				
*B. serrafreirei*	84.87	85.76*–*86.94	86.35	84.55	48.66	–			
*T. torrei*	84.27	86.35*–*87.24	86.94	86.05	48.37	87.53	–		
*L. chavezi*	49.56	49.85*–*50.45	50.14	50.74	86.35	51	50.44	–	

a Minimum values of similarity among *Lagostonema* studied populations.

b Maximum value of similarity among *Lagostonema* studied populations.

c Maximum value between *Lagostonema* studied populations and other Molineidae species.

d Minimum value between *Lagostonema* studied populations and other Molineidae species.

**Figure 6.**
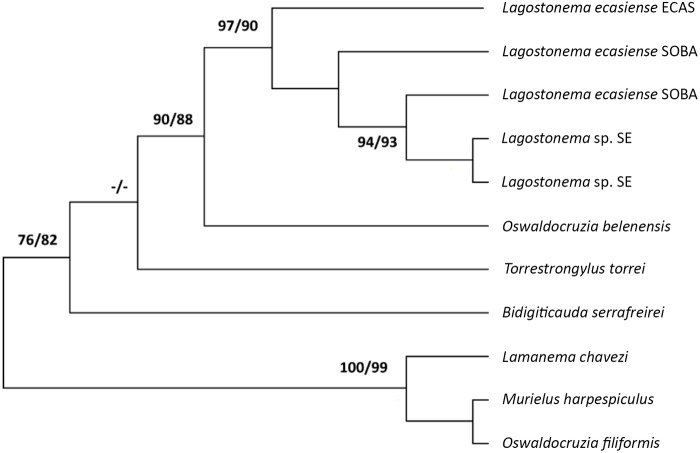
Phylogenetic tree of Molineidae species based on *cox*1 mtDNA region obtained with Maximum Likelihood (ML) and Bayesian Inference (BI).

The consensus tree showed that the haplotypes corresponding to *Lagostonema* specimens formed a clade with good resolution (97 BI/90 ML), being *Oswaldocruzia belenensis* the sister species. Considering the analysed populations of *Lagostonema*, a high genetic similarity was observed between the haplotypes of SE forming a separate subclade, following those of SOBA and ECAS (Figure 6).

## Discussion

*Lagostonema ecasiense* is a monotypic species, which has been described based on a single male (holotype) and a single female (allotype) from a host housed at the ECAS, Berazategui Department, Buenos Aires Province (Sutton and Durette-Desset, 1987). Other records also include 2 sites in La Pampa Province (Foster et al., 2002) and 2 sites in Buenos Aires Province (Canova et al., 2024). The species has not been reported from other hosts than *L. maximus*. Sutton and Durette-Desset (1987) mentioned as well the Province of Mendoza as a locality for *L. ecasiense*, although their description is based on the worms from a single individual host. This is explained by the fact that the host kept in semi-captivity in Buenos Aires was originally from Mendoza (Lunaschi et al., 2012). In these conditions, it was not possible to know whether the parasites were acquired in the province of origin of the host or the ECAS.

Although Sutton and Durette-Desset (1987) mentioned the location of *L. ecasiense* in both the stomach and the small intestine, the counting of the specimens in both organs during the present study suggests that the first portion of the small intestine is the preferred habitat for this species. The occurrence in the stomach appears secondary and may be due to a post-mortem effect, given the low abundance of the parasite in this organ.

An axis of orientation of the synlophe ridges, coincident with the sagittal axis and directed from ventral to dorsal, was observed. This contradicts the general diagnosis for the subfamily Molineinae in which it is established that the ridges are perpendicular to the body surface (Beveridge et al., 2014). In this sense, the findings of Sutton and Durette-Desset (1987) regarding the orientation of the ridges are confirmed here, placing *Lagostonema*, along with *Hugotnema* (Durette-Desset and Chabaud, 1981), within the most specialized Molineinae (Sutton and Durette-Desset, 1987).

This survey extends the geographical distribution of genus *Lagostonema* to 3 new provinces and 9 additional departments in Argentina. In addition, ecological data are provided. Previous ecological data for this species were only provided by Foster et al. (2002) in 2 populations of *L. maximus* from La Pampa Province and by Canova et al. (2024) in 2 populations from Buenos Aires Province. The P values of *Lagostonema* specimens found in ER and SE were higher than those reported by Foster et al. (2002) for La Pampa and that reported by Canova et al. (2024) in southwest Buenos Aires, and similar to that reported by Canova et al. (2024) in ECAS. However, the P recorded in this study from Buenos Aires Province was lower than those reported by Foster et al. (2002) for La Pampa; and higher than that in southwest Buenos Aires, but lower than that for ECAS reported by Canova et al. (2024). Likewise, the MIs recorded in this study were higher than that for the Toay Department but lower than that for the Caleu Caleu Department in La Pampa (Foster et al., 2002). They were also higher than those reported by Canova et al. (2024) for both sites from Buenos Aires. Finally, the MAs were higher than those reported by Canova et al. (2024) for both sites from Buenos Aires. In this sense, variations in P, MI and MA observed may be influenced by intrinsic characteristics of parasite species, environmental heterogeneity, seasonal factors and host density and immune status, among others (Arneberg et al., 1997, 1998; Poulin, 2006).

Although morphometric analysis indicated that all specimens studied belong to a single group, the SE population exhibited a tendency to diverge.

Moreover, the molecular characterization with the sequences of 9 haplotypes for the ITS1 rDNA region and 5 haplotypes for the partial gene encoding *cox*1 mtDNA provided herein constitutes the first molecular contribution for the genus *Lagostonema*. The analysis of genetic distances and phylogenetic exploration for both markers allow for consideration of *L. ecasiense* as a nominal species, confirming its nomenclatural taxonomic identity. Although morphological studies identify *Lagostonema* specimens from *L. maximus* populations as only one species, molecular studies reveal a significant genetic distance separating the SE population from the others.

In this framework, until more evidence is obtained from morphological studies and new molecular markers, the SE haplotypes are mentioned as *Lagostonema* sp., considering it a possible cryptic species or divergent population (species in the process of speciation).

In this context, it should be mentioned that animal populations in the SE area have suffered significant environmental fragmentation, including *L. maximus* populations that have been isolated by a significant landscape transformation (Correa et al., 2012; Aguiar et al., 2016). Studying their parasites could provide insight into the bioecological and evolutionary characteristics of the parasite–host association (Poulin, 2004).

It is important to highlight that no molecular information exists for the parasite species found and described for *L. maximus* except for what was reported by Canova et al. (2021) and Canova et al. (2023). Therefore, this bias in the available information shows the relevance of providing new sequences from these taxonomic groups with little genetic representation.

This study is valuable because it contributes to the ratification of a nominal species described decades ago, adding new morphological aspects and providing an understanding of their value as a marker of host populations, mainly in species of ecological, health and economic importance such as *L. maximus* in Argentina.

## Supporting information

Canova et al. supplementary material Canova et al. supplementary material

## Supplementary material

The supplementary material for this article can be found at https://doi.org/10.1017/S0031182025100528.
