# Supplementary figures and images for: Morphological data and molecular characterization of Lagostonema ecasiense (Nematoda, Molineidae) parasite of Lagostomus maximus (Rodentia, Chinchillidae) from Argentina and other considerations
Source: Parasitology. 2025 Jul 14;152(9):918–31. doi: 10.1017/S0031182025100528 (PMC12644954; doi:10.1017/S0031182025100528)

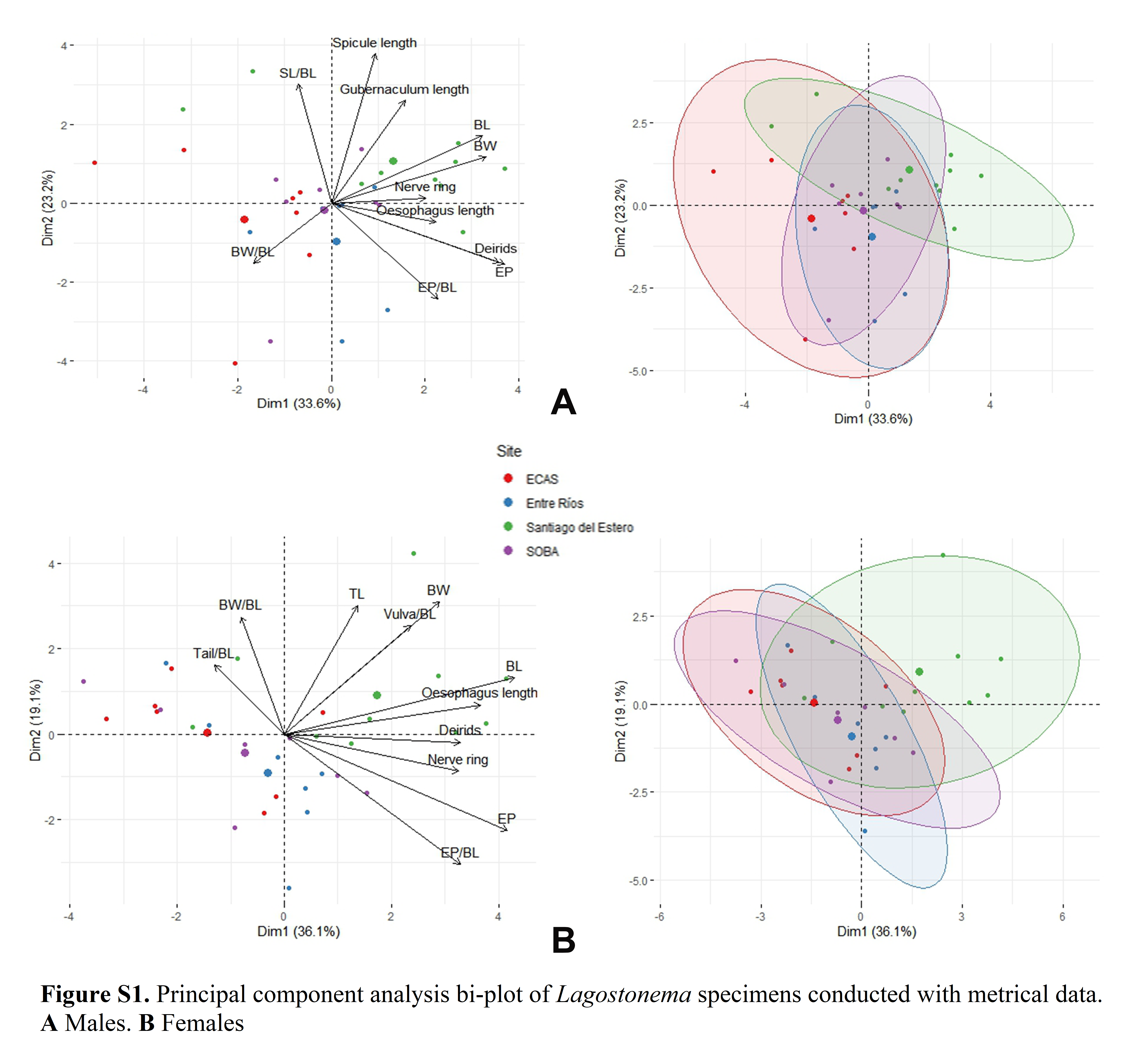

Supplement: Canova et al. supplementary material [file S0031182025100528sup001.tiff]
